# FDA and EMA-approved noninvasive imaging techniques for basal cell carcinoma subtyping: A systematic review

**DOI:** 10.1016/j.jdin.2025.05.006

**Published:** 2025-06-10

**Authors:** Mehdi Boostani, Giovanni Pellacani, Ximena Wortsman, Mariano Suppa, Mohamad Goldust, Carmen Cantisani, Paweł Pietkiewicz, Kende Lőrincz, András Bánvölgyi, Norbert M. Wikonkál, Wendy J. Huss, Kamran Avanaki, Gyorgy Paragh, Norbert Kiss

**Affiliations:** a Department of Dermatology, Roswell Park Comprehensive Cancer Center, Buffalo, New York; b Department of Dermatology, Venereology and Dermatooncology, Semmelweis University, Budapest, Hungary; c Department of Dermatology, Sapienza University of Rome, Rome, Italy; d Department of Dermatology, Faculty of Medicine, Universidad de Chile, Santiago, Chile; e Department of Dermatology and Cutaneous Surgery, Miller School of Medicine, University of Miami, Miami, Florida; f Groupe d'Imagerie Cutanée Non-Invasive (GICNI) of the Société Française de Dermatologie (SFD), Paris, France; g Department of Dermatology, Hôpital Erasme, Université Libre de Bruxelles, Brussels, Belgium; h Department of Dermatology, Institut Jules Bordet, Université Libre de Bruxelles, Brussels, Belgium; i Department of Dermatology, Yale University School of Medicine, New Haven, Connecticut; j Centrum Medyczne Zwierzyniecka, Poznań, Poland; k Unitelematica Leonardo da Vinci, Zug, Switzerland; l Central Hospital of Northern Pest–Military Hospital, Budapest, Hungary; m Richard and Loan Hill Department of Biomedical Engineering, University of Illinois at Chicago, Chicago, Illinois; n Department of Dermatology, University of Illinois at Chicago, Illinois

**Keywords:** basal cell carcinoma, dermoscopy-guided high-frequency ultrasound, dynamic optical coherence tomography high-definition optical coherence tomography, imaging, high-frequency ultrasound, line-field confocal optical coherence tomography, noninvasive, optical coherence tomography, reflectance confocal microscopy, subtype, subtyping

## Abstract

**Background:**

Basal cell carcinoma (BCC), the most common skin cancer, requires accurate subtyping for optimal treatment. While histopathology is the gold standard, noninvasive imaging offers a biopsy-free alternative, though its accuracy remains unclear.

**Objective:**

To assess the diagnostic accuracy of Food and Drug Administration- and European Medicines Agency-approved noninvasive imaging techniques for BCC subtyping.

**Methods:**

A systematic review included 19 studies (2650 lesions) from EMBASE, Scopus, PubMed, and Cochrane Library (published up to November 30, 2024). Evaluated modalities included dermoscopy, high-frequency ultrasound (HFUS), dermoscopy-guided HFUS (DG-HFUS), optical coherence tomography (OCT), high-definition OCT (HD-OCT), dynamic OCT (D-OCT), line-field confocal OCT (LC-OCT), and reflectance confocal microscopy.

**Results:**

Dermoscopy showed moderate accuracy (81.9% sensitivity, 81.8% specificity for superficial BCC [sBCC]). DG-HFUS outperformed HFUS (82.4% sensitivity, 91.3% specificity). LC-OCT had the highest accuracy, achieving 100% sensitivity for infiltrative BCC. D-OCT distinguished subtypes at 150 μm depth; branching vessels at 300 μm increased nBCC risk (relative risk = 1.53, *P* = .016). HD-OCT had 100% sensitivity for sBCC but lower accuracy for other subtypes. Reflectance confocal microscopy had 88.9% sensitivity for non-sBCC but only 33.3% for aggressive subtypes.

**Limitations:**

Study heterogeneity and inconsistent protocols limit comparability.

**Conclusion:**

LC-OCT and HD-OCT offer the highest accuracy, while DG-HFUS outperforms dermoscopy and HFUS, making them strong noninvasive alternatives.

**Capsule Summary**

• Histopathology is the gold standard for basal cell carcinoma subtyping, but noninvasive imaging techniques have been explored with variable accuracy.
• Line-field confocal optical coherence tomography and high-definition optical coherence tomography provide superior accuracy for basal cell carcinoma subtyping.

## Introduction

Basal cell carcinoma (BCC) is the most prevalent skin cancer and the most common malignancy among fair-skinned populations.^1^ Current guidelines categorize BCC into several histopathologic subtypes: superficial BCC (sBCC), nodular BCC (nBCC), infiltrative BCC (iBCC), micronodular BCC (mnBCC), sclerodermiform BCC, basosquamous BCC, keratotic variant, infundibulocystic variant, fibroepithelioma of Pinkus and mixed. Among these, iBCC, mnBCC, sclerodermiform BCC, and basosquamous BCC are considered aggressive due to their higher risk of recurrence and deeper tissue invasion. In contrast, sBCC and nBCCs have a lower likelihood of deep tissue infiltration. Additionally, nonaggressive subtypes include the keratotic variant, infundibulocystic variant, and fibroepithelioma of Pinkus. Diagnostic accuracy for detection of BCC by dermoscopy alone is generally >98%, but biopsies are typically performed in order to categorize the BCC by subtype, because subtypes exhibit distinct biological behaviors, prognoses, and recurrence risks, influencing therapeutic strategies.^2^ Accurate preoperative classification of BCC subtypes is essential for selecting the most effective treatment modality, as different subtypes require distinct management approaches.^3^ sBCC, for instance, can often be treated noninvasively with topical therapies, cryotherapy, or superficial excision, while nBCC typically requires standard surgical excision due to its deeper dermal involvement. iBCC, being more aggressive with a higher risk of recurrence, often requires Mohs micrographic surgery to ensure complete removal while preserving healthy tissue.^3^ Therefore, precise subtype identification guides clinicians in choosing the most appropriate and effective treatment strategy. Advanced preoperative tools, such as multispectral imaging,^4^ high-frequency ultrasound (HFUS),^4^, ^5^, ^6^, ^7^ optical coherence tomography (OCT),^8^ and reflectance confocal microscopy (RCM),^9^ among others, have potential to complement or replace histological sampling by offering real-time evaluation, allowing for immediate clinical decision-making.^10^ However, the utility of imaging for BCC subtyping remains inadequately explored. This systematic review aims to evaluate Food and Drug Administration (FDA)- and European Medicines Agency (EMA)-approved imaging techniques, focusing on their ability to distinguish BCC subtypes.

## Materials and methods

### Literature search

This systematic review was registered on international prospective register of systematic reviews (No: CRD42025628817) and conducted in accordance with Preferred Reporting Items for Systematic Reviews and Meta-Analyses guidelines.^11^ A structured literature search was performed on November 26, 2024, across EMBASE, Scopus, PubMed, and Cochrane databases using targeted keywords and Boolean operators (eg, AND, OR) to refine results. Search terms included “basal AND cell AND carcinoma” AND (subtype OR subtypes OR type OR types OR discriminating OR subtyping OR diagnosis OR diagnostic OR diagnosing OR descriptive OR classifying OR invasive) AND (“dermatoscopy” OR “dermoscopy” OR “optical coherence tomography” OR “reflectance confocal microscopy” OR “laser scanning confocal microscopy” OR “ultrasound”). Given the heterogeneity in study methodologies and reported outcomes, a Synthesis Without Meta-Analysis approach was employed.^12^

### Selection criteria

The population, index test, reference standard, diagnosis of interest framework structured the study's clinical question. The inclusion criteria were as follows:

P (Population): Individuals diagnosed with BCC.

I (Index test): Noninvasive imaging technologies approved by the FDA or EMA, including DG-HFUS, HFUS, OCT, D-OCT, line-field confocal optical coherence tomography (LC-OCT), and RCM.

R (Reference standard): All types of histopathologic evaluation were accepted as reference standard. When more than one type of histopathologic evaluation was available, excisional biopsy with serial sections was chosen.

D (Diagnosis of interest): BCC subtype.

Selected studies included observational, retrospective, prospective, case series, and randomized designs meeting population, index test, reference standard, diagnosis of interest criteria. Exclusion criteria were pediatric, preclinical, animal studies, reviews, abstracts, case reports, non-English publications, and studies lacking relevant data or full text.

### Quality assessment

The methodological quality of included studies was assessed using the Quality Assessment of Diagnostic Accuracy Studies-2 tool by 2 independent authors (M.B. and N.K.). Four domains, patient selection, index test, reference standard, and flow/timing, were evaluated. Studies with low risk in all domains were classified as low risk; others were considered at risk.

## Results

### Identification of approved imaging techniques

A total of 8 imaging modalities have been identified, 6 of which are approved by both the FDA and the EMA for the assessment of BCC. These include dermoscopy, HFUS, OCT, dynamic optical coherence tomography (D-OCT), high-definition optical coherence tomography (HD-OCT) and RCM. Table I provides an overview of these imaging techniques.

**Table I:** Overview of the imaging techniques used in basal cell carcinoma subtyping

Imaging technique	Visualization depth	Resolution	Source of contrast	Technique description	Example devices
Dermoscopy^13^, ^14^, ^15^, ^16^, ^17^	Surface only	∼10-30 μm	Skin discoloration (may be due to concentrations of melanin, hemoglobin, and keratin absorption and scattering at the skin surface).	Noninvasive surface imaging for analyzing pigmented skin lesions.	• Heine DELTA 30 by Heine Optotechnik (Germany)^18^ • Dermlite DL4 by Dermlite (USA)^19^ • Illuco IDS 1100 by Illuco (South Korea)^20^
HFUS^21^, ^22^, ^23^, ^24^, ^25^, ^26^ (including DG-HFUS^5^)	1-10's of mms	40-200 μm	Acoustic impedance mismatches between microstructures in tissue causing ultrasound scattering and reflection.	Noninvasive ultrasound for structural imaging of epidermis, dermis and subcutaneous tissues. In DG-HFUS, images are co-registered with dermoscopy.	• SkinScanner by Dermus (Hungary)^27^ • Vevo MD by VisualSonics (Canada)^28^ • DermaScan C by Cortex Technology (Denmark)^29^
OCT^30^, ^31^, ^32^, ^33^, ^34^ (all types)	1-2 mm	<15 μm	Changes in optical scattering due to variations in refractive index and tissue microstructures.	Noninvasive imaging method for high-resolution volumetric, en face, or cross-sectional tissue analysis.	• VivoSight by Michelson Diagnostics (United Kingdom)^35^
HD-OCT^36^	Up to 1 mm	3 μm	Same as OCT.	Same as OCT. Provides near-cellular resolution.	• Skintell® by Agfa Healthcare (Belgium)^37^
D-OCT^38^^,^^39^	1-2 mm	10s of μm	Temporal changes in optical scattering of blood.	Functional imaging of blood flow.	• VivoSight Dx by Michelson Diagnostics (United Kingdom)^40^
LC-OCT^41^, ^42^, ^43^, ^44^	500 μm	1-3 μm	Same as OCT.	Combines confocal microscopy with OCT principles for high-resolution en face and cross-sectional imaging, suitable for analyzing skin with near-cellular-level detail.	• deepLive by Damae Medical (France)^45^
RCM^46^, ^47^, ^48^	200-300 μm	∼1 μm	Differences in refractive index between cellular structures, which leads to variations in backscattering of light.	High-resolution imaging for near-cellular-level analysis, particularly for diagnosing skin cancer by observing cell morphology and arrangement.	• VivaScope by VivaScope GmbH (Germany)^49^

*DG-HFUS*, Dermoscopy-guided high-frequency ultrasound; *D-OCT*, dynamic optical coherence tomography; *HFUS*, high-frequency ultrasound; *HD-OCT*, high-definition optical coherence tomography; *LC-OCT*, line-field confocal optical coherence tomography; *OCT*, optical coherence tomography; *RCM*, reflectance confocal microscopy.

### Selection of studies

**Fig 1:**
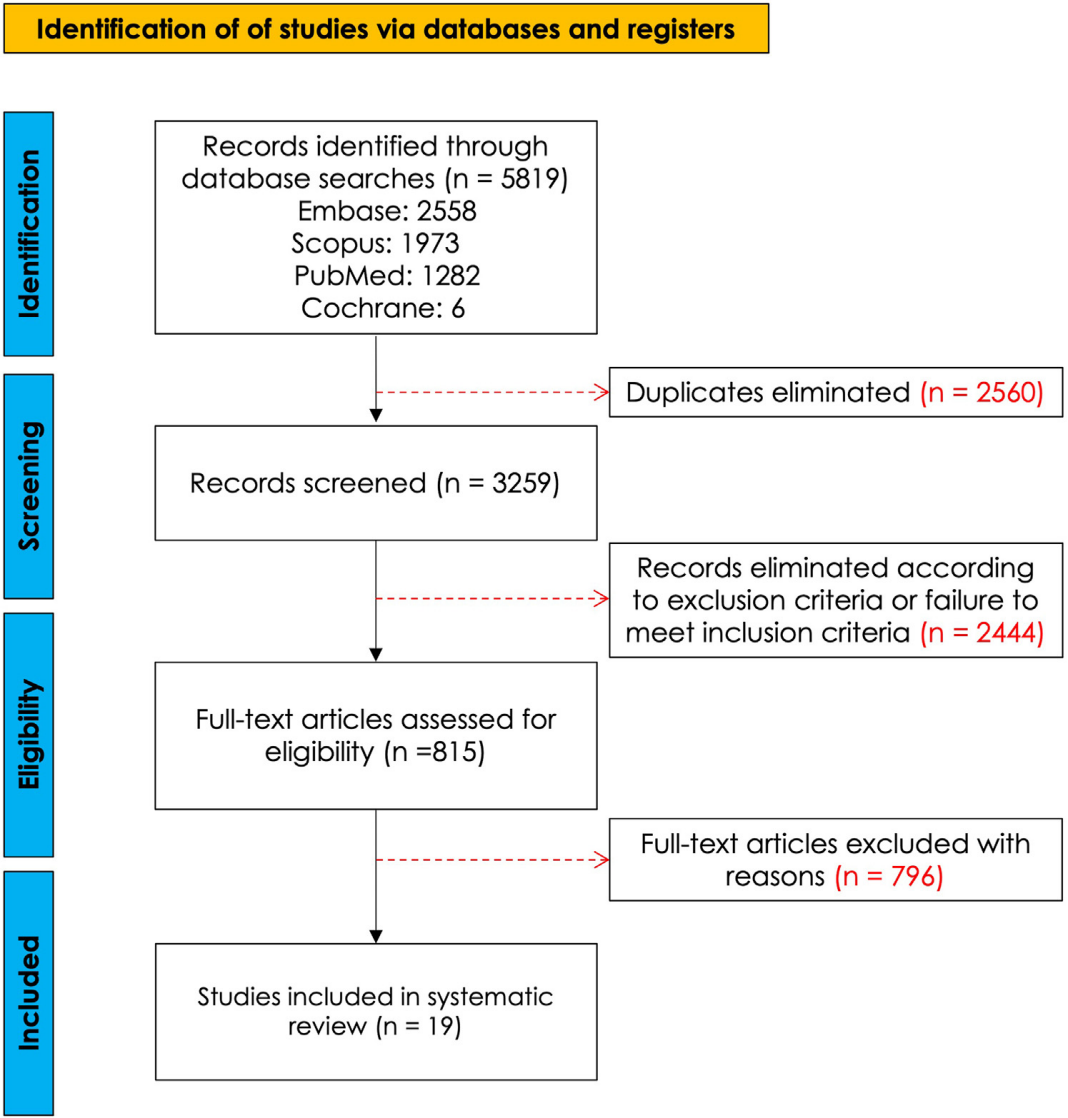
PRISMA flow diagram illustrating the screening and selection process conducted according to PRISMA guidelines. This diagram outlines the number of studies identified through database searches, the number of records screened, those excluded, and the final number of studies included in the review, as per the PRISMA framework for scoping reviews. 10 PRISMA, Preferred Reporting Items for Systematic Reviews and Meta-Analyses. A systematic search across multiple databases identified a total of 5819 records, retrieved from Embase 2558, Scopus 1973, PubMed 1282, and Cochrane 6. After removing 2560 duplicates, 3259 unique records were screened for relevance. Of these, 2444 were excluded as they did not meet the predefined inclusion criteria outlined in Selection criteria. Following further assessments, the pool was narrowed to 815 full-text articles, which were then thoroughly reviewed. This process led to the exclusion of 796 articles that either did not meet the inclusion criteria or fell under the exclusion criteria. Ultimately, 19 studies met the specific scoping criteria and were included in this systematic scoping review.

A systematic search across multiple databases identified 5819 records, and after screening and review, 19 studies met the criteria for inclusion (Fig 1).

### Study characteristics

This systematic review analyzed 19 studies published between 2012 and 2023, comprising a total of 2650 lesions. Europe contributed 18 studies, and Asia one. Dermoscopy (*n* = 5) assessed 1207 lesions, HFUS (*n* = 3) assessed 399 lesions, DG-HFUS (*n* = 1) evaluated 63 lesions, OCT (*n* = 3) examined 783 lesions, HD-OCT (*n* = 1) studied 48 lesions, D-OCT (*n* = 1) evaluated 98 lesions, LC-OCT (*n* = 1) assessed 52 lesions, and RCM (*n* = 4) included 383 lesions.

### Study quality

The risks of bias and applicability concerns for the included studies, assessed using the Quality Assessment of Diagnostic Accuracy Studies-2 tool, showed low bias in most studies, particularly in the reference standard and flow/timing domains. However, a few studies exhibited higher bias in the patient selection domain, raising applicability concerns due to small sample sizes or limited generalizability (Table II). All studies, regardless of their risk of bias, were included in the analysis.

**Table II:** Risk of bias and applicability concerns for included studies

Author, y	Risk of bias					Applicability concern			
	Patient selection	Index test	Reference standard	Flow and timing	Overall risk of bias	Patient selection	Index test	Reference standard	Overall applicability concern
Lallas et al, 2014	Low	Low	Low	Low	Low	Low	Low	Low	Low
Pampena et al, 2021	Low	Low	Low	Low	Low	Low	Low	Low	Low
Popadić et al, 2014	Low	Low	Low	Low	Low	Low	Low	Low	Low
Popadić et al, 2022	Low	Low	Low	Low	Low	Low	Low	Low	Low
Trigoni et al, 2012	Low	Low	Low	Low	Low	Low	Low	Low	Low
Bozsányi et al, 2023	Low	Low	Low	Low	Low	Low	Low	Low	Low
Hernández-Ibáñez et al, 2017	Low	Low	Low	Low	Low	Low	Low	Low	Low
Siskou et al, 2023	Low	Low	Low	Low	Low	Low	Low	Low	Low
Wang et al, 2021	Low	Low	Low	Low	Low	Low	Low	Low	Low
Holmes et al, 2018	Low	Low	Low	Low	Low	Low	Low	Low	Low
Sinx et al, 2020	Low	Low	Low	Low	Low	Low	Low	Low	Low
Adan et al, 2021	Low	Low	Low	Low	Low	Low	Low	Low	Low
Boone et al, 2016	High	Low	Low	Low	High	High	Low	Low	High
Themstrup et al, 2017	Low	Low	Low	Low	Low	Low	Low	Low	Low
Ruini et al, 2021	Low	Low	Low	Low	Low	Low	Low	Low	Low
Longo et al, 2014	Low	Low	Low	Low	Low	Low	Low	Low	Low
Peppelman et al, 2013	High	Low	Low	Low	High	High	Low	Low	High
Lupu et al, 2019	Low	Low	Low	Low	Low	Low	Low	Low	Low
Woliner–van der Weg et al, 2021	Low	Low	Low	Low	Low	Low	Low	Low	Low

Based on the Quality Assessment of Diagnostic Accuracy Studies-2 (QUADAS-2) tool, most studies reported low risk of bias for patient selection, reference standard, and flow and timing. Of the 20 studies included, 18/20 were classified as having a low overall risk of bias, and 18/20 had low overall applicability concerns. Despite this, some studies were classified with high risk.

### Diagnostic modalities in BCC subtyping

#### Dermoscopy

Lallas et al^50^ reported that dermoscopy had a sensitivity of 81.9% and specificity of 81.8% for subtyping sBCC (Table III). Pampena et al^51^ demonstrated that dermoscopy improved diagnostic accuracy, with arborizing telangiectasia being most common in nBCC (81.8%), compared to iBCC (71.8%) and sBCC (6.7%). Short fine superficial telangiectasia was most prevalent in sBCC (74.8%), whereas it was observed in only 3.2% of nBCCs and 19.7% of iBCCs. Popadić et al^52^^,^^53^ found high sensitivity values for key dermoscopic features, with sBCC showing leaf-like areas (98.2%), short fine telangiectasia (61.4%), and structureless hypopigmentation (54.4%). nBCC was associated with a milky red background (76.7%), arborizing vessels (53.3%), and microvessels (51.7%). For aggressive BCC subtypes, ulceration had 100% sensitivity, while annular telangiectatic vessels (90.5%), milky red background (76.2%), and arborizing microvessels (66.7%) were also significant indicators. Their 2022 study confirmed excellent subtyping for sBCC (κ = 0.85), with a sensitivity of 93.9% for white-red unstructured areas and a specificity of 97.1% for short fine telangiectasia. For nBCC, sensitivity for white-red areas was 89.1%, while specificity for blue-gray nests reached 94.6%, with a positive predictive value (PPV) of 76.9% for blue-gray nests and a negative predictive value of 58.3% for ulceration. In aggressive BCC subtypes, sensitivity for white-red areas was 82.6%, and specificity for blue-gray nests was 87.3%. Trigoni et al^54^ identified significant dermoscopic markers for pigmented and nonpigmented BCCs.

**Table III:** Characteristics of the included studies

Author	Year	Country	Noninvasive skin imaging tools	Total patients	Mean patient age (y)	No. (%) of patients by sex	Lesions (*n*)	Design	Objective	Outcome
Trigoni et al^54^	2012	Greece	Dermoscopy	96	NA	60 (62.5%) M 36 (37.5%) F	138	Retrospective observational	To identify and statistically evaluate the dermoscopic findings of pigmented and non-pigmented nodular and sBCCs, and assess their significance.	The study found that sBCCs were more likely to show comma vessels (93%), white-red structureless areas (90%), hypopigmented areas (64%), and small ulcerations (79%).
Lallas et al^50^	2014	Italy	Dermoscopy	313	67.1 ± 15.3	172 (55% M) 141 (45% F)	335	Retrospective observational	To evaluate the diagnostic accuracy of dermoscopic criteria for distinguishing sBCC from other BCC subtypes.	Dermoscopy demonstrated 81.9% sensitivity and 81.8% specificity for distinguishing sBCC from other BCC subtypes, with key predictors identified for sBCC and non-sBCC lesions.
Popadić et al^52^	2014	Serbia	Dermoscopy	116	67.7	64 (55.2%) M 52 (44.8%) F	151	Prospective observational	To compare dermoscopic features across different morphologic types of BCC.	Dermoscopy revealed sensitivity values for sBCC features such as leaf-like areas (98.2%), short fine telangiectasias (61.4%), and structureless hypopigmentation (54.4%).
Pampena et al^51^	2021	Italy	Dermoscopy	481	65	241 (50.1%) M 240 (49.9%) F	481	Retrospective observational	To identify clinical and dermoscopic criteria to differentiate iBCC from sBCC and nBCC.	Dermoscopy improved diagnostic accuracy, increasing sensitivity, specificity, and predictive values for differentiating among all BCC subtypes, including sBCC.
Popadić et al^53^	2022	Serbia	Dermoscopy	62	66.4	35 (44%) M 27 (56%) F	102	Retrospective observational	To evaluate the accuracy of dermoscopy in diagnosing different subtypes of BCC.	Dermoscopy showed excellent diagnostic accuracy for sBCC with kappa = 0.85, sensitivity of 93.9% for white-red unstructured areas, and specificity of 97.1% for short fine telangiectasias.
Bozsányi et al^6^	2023	Hungary	DG-HFUS	60	73.1 ± 10.6	34 (56.7%) M 26 (43.3%) F	63	Prospective observational	To evaluate the ability of DG-HFUS to identify aggressive BCC subtypes and aid early treatment planning, using a 3-step algorithm to differentiate low- and high-risk subtypes.	DG-HFUS identified aggressive BCC subtypes with higher sensitivity (82.4%) and specificity (91.3%) than dermoscopy. The diagnostic algorithm demonstrated superior positive predictive value (94.7%) and negative predictive value (78.6%) for sBCCs.
Hernández-Ibáñez et al^55^	2017	Spain	HFUS	156	68 ± 12	92 (59%) M 64 (41%) F	156	Retrospective observational	To compare the diagnostic accuracy of HFUS compared with punch biopsy in classifying histologic subtypes of BCC.	HFUS showed a positive predictive value (PPV) of 93.3% for sBCCs, comparable to punch biopsy (92%).
Wang et al^56^	2021	China	HFUS	100	47%	47 (47%) M 53 (53%) F	143	Retrospective and prospective validation cohort	To develop a predictive model utilizing HFUS features for accurate differentiation of BCC subtypes.	HFUS-based predictive model showed 76.7% accuracy for sBCC, and invasive BCCs exhibited irregular growth patterns.
Siskou et al^57^	2023	Greece	HFUS	54	NA	66 (66%) M 34 (34%) F	100	Retrospective observational	To evaluate HFUS in distinguishing high-risk from low-risk BCC subtypes, by correlating ultrasound features with histologic results.	HFUS demonstrated almost perfect agreement with histology for classifying BCC subtypes, with Cohen's kappa of 0.8251 for sBCCs.
Holmes et al^58^	2018	United Kingdom	OCT	NA	NA	NA	234	Prospective observational	To evaluate the impact of diagnostic criteria, image quality, lesion location, observer confidence, and interobserver variability on the diagnostic performance of OCT, and to assess its potential for diagnosing BCC subtypes.	OCT predicted sBCC with 78% accuracy, distinguishing it by the presence of dark dermal borders and bulges extending into the dermis.
Sinx et al^59^	2020	Netherlands	OCT	182	66.8 ± 13	93 (51.1%) M 83 (45.6%) F	250	Prospective cohort	To assess the ability of OCT to differentiate between sBCC and non-sBCC subtypes.	OCT combined with dermoscopic images showed a significant increase in specificity for sBCC, from 47.8% by dermoscopic examination alone to 78.3%.
Adan et al^60^	2021	Netherlands	OCT	299	72	164 (54.8%) M 135 (45.2%) F	299	Prospective cohort	To evaluate the diagnostic value of OCT image features for identifying and subtyping BCC.	OCT identified sBCC with 84.3% positive predictive value, demonstrating high accuracy in BCC subtyping without the need for biopsy in 44% of cases.
Boone et al^61^	2016	Belgium	HD-OCT	48	NA	20 (41.7%) M 28 (58.3%) F	48	Retrospective pilot	To determine the optimal critical value for distinguishing BCC subtypes using HD-OCT by quantifying light attenuation and other *in vivo* optical properties.	HD-OCT demonstrated 97.9% diagnostic accuracy for sBCCs, with sensitivity and specificity of 100% and 96.87%, respectively.
Themstrup et al^62^	2017	Denmark	D-OCT	81	67 ± 14.8	46 (57%) M 35 (43%) F	98	Prospective observational	To evaluate D-OCT's effectiveness in distinguishing BCC subtypes using microvascular and structural imaging.	D-OCT distinguished sBCC based on vascular mottle patterns (100% in sBCC) and serpinginous vessels (78% reduced risk for sBCC).
Ruini et al^63^	2021	Germany	LC-OCT	52	71	35 (67%) M 17 (33%) F	52	Prospective cohort	To evaluate the key LC-OCT criteria for diagnosing and subtyping BCC in comparison with histopathology, OCT, and RCM.	LC-OCT showed 82% sensitivity and 100% specificity for sBCC, with an AUC of 0.98.
Peppelman et al^64^	2013	Netherlands	RCM	27	65.7 ± 10	16 (59%) M 11 (41%) F	27	Prospective observational	To establish RCM features associated with different BCC subtypes.	RCM identified specific features of sBCC, including tumor nest location beneath the basal layer and solar elastosis.
Longo et al^65^	2014	Italy	RCM	88	60.6 ± 14.5	NA	88	Retrospective Observational	To identify and define specific confocal criteria for distinguishing between different BCC subtypes.	RCM showed that the presence of cords connected to the epidermis was a highly significant predictor for sBCC (OR 54.63, *P* < 0.001).
Lupu et al^66^	2019	Romania	RCM	87	68.1 ± 12.17	36 (41.3%) M 51 (58.7%) F	123	Retrospective observational	To assess the accuracy of RCM criteria in classifying BCC subtypes and evaluate intraobserver reliability.	RCM demonstrated high accuracy for subtyping BCC, with key features predicting sBCC (cords connected to the epidermis, OR = 6.79) and aggressive BCC (hyporefractile silhouettes, OR = 16.92).
Woliner–van der Weg et al^67^	2021	Netherlands	RCM	145	NA	78 (53.8%) M 67 (46.2%) F	145	Randomized clinical trial	To assess the diagnostic accuracy of RCM for subtyping BCC compared to punch biopsy.	RCM was effective for subtyping non-sBCC (88.9% sensitivity) but had low sensitivity for aggressive BCC (33.3%).

*3D HD-OCT*, Three-Dimensional High-Definition Optical Coherence Tomography; *AK*, actinic keratosis; *AUC*, area under the curve; *BCC*, basal cell carcinoma; *D-OCT*, dynamic optical coherence tomography; *DG-HFUS*, dermoscopy-guided high-frequency ultrasound; *HD-OCT*, high-definition optical coherence tomography; *HFUS*, high-frequency ultrasound; *iBCC*, infiltrative basal cell carcinoma; *LC-OCT*, line-field optical coherence tomography; *NA*, not available; *NPV*, negative predictive value; *nBCC*, nodular basal cell carcinoma; *OCT*, optical coherence tomography; *OR*, odds ratio; *pBCC*, pigmented basal cell carcinoma; *PPV*, positive predictive value; *RCM*, reflectance confocal microscopy; *RR*, relative risk; *sBCC*, superficial basal cell carcinoma.

#### HFUS

Hernández-Ibáñez et al^55^ achieved a diagnostic yield of 73.7% for BCC subtyping, with a sensitivity of 74.5%, specificity of 73%, and a notably high PPV of 93.3% for sBCC. Siskou et al^57^ demonstrated almost perfect agreement with histology (Cohen's Kappa = 0.82) for sBCC classification. HFUS revealed a strong association between BCC subtypes and tumor shape, with 72.8% of iBCCs presenting an irregular shape and 82.6% of sBCCs displaying a ribbon shape (*P* = 0.000). Wang et al^56^ identified that 60.5% of invasive BCCs exhibited irregular growth patterns, while 89.5% of noninvasive BCCs were either nBCCs or showed a crawling growth pattern (*P* < 0.001).

#### DG-HFUS

Bozsányi et al^6^ found that DG-HFUS demonstrated higher diagnostic accuracy than dermoscopy, with a sensitivity of 82.4% and specificity of 91.3%, compared to dermoscopy's 40.1% sensitivity and 73.1% specificity. Validation on an independent dataset confirmed 83.33% sensitivity and 91.66% specificity.

#### OCT

Holmes et al^58^ demonstrated an accuracy of 78% for distinguishing sBCC using OCT. Additionally, the study reported 68% accuracy for nBCC. Sinx et al^59^ found that combining OCT with clinical images improved diagnostic performance, increasing the area under the curve (AUC) from 85.6% to 91.2% and raising the PPV to 89.1%. OCT combined with dermoscopic images also significantly improved specificity for sBCC, increasing from 47.8% with dermoscopic examination alone to 78.3%. Adan et al^60^ demonstrated that an OCT-based diagnostic algorithm detected 97.8% of BCCs, with 44% of cases accurately subtyped without the need for biopsy. The algorithm achieved a PPV of 84.3% for sBCC and 98.8% for non-sBCC.

#### HD-OCT

Boone et al^61^ conducted a retrospective pilot study with 48 lesions to determine the optimal criteria for distinguishing BCC subtypes using HD-OCT. The study demonstrated a high overall diagnostic accuracy of 97.9% for sBCC. Sensitivity was 100% for sBCC, 93.8% for nBCC, and 93.8% for iBCC, while specificity was 96.87% for sBCC, 96.9% for nBCC, and 93.8% for iBCC.

#### D-OCT

Themstrup et al^62^ demonstrated that D-OCT differentiated BCC subtypes by assessing vascular patterns and structural features at a depth of 150 micrometers. Key biomarkers identified for BCC subtypes included the vascular mottle pattern, which was observed in 100% of sBCC cases, 98% of nBCC, and 94% of iBCC cases. Dots appeared in 48% of sBCC cases, 51% of nBCC cases, and 63% of iBCC cases. Arborizing vessels were more frequently observed in iBCC (19%) than in sBCC (11%) or nBCC (5%). Serpiginous vessels were rare in all subtypes except nBCC, where they were present in 16% of cases, compared to 7% in sBCC and none in iBCC. Lines were more commonly seen in nBCC and iBCC (55%) compared to sBCC (30%). Circumscribed areas were found in 7% of nBCCs.

#### LC-OCT

Ruini et al^63^ demonstrated high diagnostic accuracy across BCC subtypes. For nBCC, LC-OCT showed 96% sensitivity, 96% specificity, and an AUC of 0.96. sBCC had a sensitivity of 82%, specificity of 100%, and an AUC of 0.98. iBCC exhibited 100% sensitivity, 98% specificity, and an AUC of 0.92. The mixed nBCC-sBCC subtype showed 91% sensitivity, 95% specificity, and an AUC of 0.90. Additionally, LC-OCT demonstrated 90.4% agreement with histology.

#### RCM

Longo et al^65^ demonstrated that RCM accurately identified different BCC subtypes. The presence of epidermal cords strongly predicted sBCC (odds ratio [OR] = 54.63, *P* < 0.001), while clefting was linked to nBCC (OR = 17.13, *P* < 0.001). iBCC was associated with tumor islands (OR = 0.131, *P* = 0.030) and the absence of epidermal cords (OR = 0.025, *P* < 0.001). Peppelman et al^64^ identified key biomarkers for BCC subtypes. Solar elastosis was present in 100% of sBCCs, but less frequent in nBCC (18.2%) and mnBCC (33.3%). Conversely, fibrosis surrounding tumor nests was highly prevalent in nBCC (100%), but occurred less frequently in sBCC (42.9%) and mnBCC (66.7%). Lupu et al^66^ reported an overall diagnostic accuracy of 97.1% sensitivity and 78.95% specificity for BCC detection using RCM. For subtyping, epidermal cords (54.2%) and peripheral palisading (79.2%) were key biomarkers for sBCC, while large tumor islands (75.4%) and increased vascularization (75.4%) were indicative of nBCC. Woliner–van der Weg et al^67^ found that RCM exhibited 88.9% sensitivity for identifying non-sBCC subtypes, but sensitivity was significantly lower at 33.3% for detecting aggressive BCC subtypes.

## Discussion

### Comparative analysis

DG-HFUS performed better in distinguishing low-risk and high-risk BCC, with 82.4% sensitivity and 91.3% specificity.^6^ HD-OCT and LC-OCT demonstrated high diagnostic accuracy. HD-OCT achieved 97.9% accuracy for sBCC, with sensitivity of 100% for sBCC, 93.8% for nBCC, and 93.8% for iBCC, and specificity of 96.87% for sBCC, 96.9% for nBCC, and 93.8% for iBCC. LC-OCT showed sensitivity of 96% and specificity of 96% for nBCC (AUC 0.96); sensitivity of 82% and specificity of 100% for sBCC (AUC 0.98); and sensitivity of 100% and specificity of 98% for iBCC (AUC 0.92),^63^ outperforming RCM in overall accuracy (90.4% agreement with histology). Nevertheless, RCM, showed promising results, particularly in detecting sBCC with an OR of 54.6 (*P* < 0.001) for cords connected to the epidermis and nBCC in the presence of clefting with an OR of 17.1 (*P* < 0.001).^65^ While dermoscopy remains valuable for initial detection, advanced techniques like DG-HFUS, HD-OCT, LC-OCT and RCM demonstrate superior diagnostic accuracy and sensitivity for BCC subtyping.

HD-OCT and LC-OCT both exhibited remarkable diagnostic performance for BCC subtyping, yet they differed in specific strengths. For sBCC, HD-OCT demonstrated superior sensitivity, achieving 100% sensitivity and 96.87% specificity, with an overall accuracy of 97.9%.^61^ This ensured a high degree of confidence in ruling out false negatives. In comparison, LC-OCT achieved slightly lower sensitivity at 82% but compensated with a perfect specificity of 100% and an AUC of 0.98.^63^ For nBCC, HD-OCT and LC-OCT exhibited comparable accuracy, with HD-OCT achieving 93.8% sensitivity, 96.9% specificity, and 95.83% overall accuracy. LC-OCT demonstrated a slightly higher sensitivity of 96%, with a matching specificity of 96% and an AUC of 0.96. Moreover, LC-OCT showed excellent agreement with histology, achieving a kappa value of 0.92 for nBCC. While both modalities performed exceptionally well for this subtype, LC-OCT's higher sensitivity and robust histological agreement enhanced its diagnostic reliability.

LC-OCT achieved perfect sensitivity of 100% and specificity of 98% for iBCC, with an AUC of 0.92, demonstrating its clear advantage in detecting this subtype. While still highly effective, HD-OCT recorded slightly lower metrics, with 93.8% sensitivity, 93.8% specificity, and an accuracy of 93.8%. LC-OCT further stood out in its ability to diagnose mixed nBCC-sBCC, achieving 91% sensitivity, 95% specificity, and an AUC of 0.90. HD-OCT lacked reported data on mixed BCC. Overall, HD-OCT excelled in sensitivity, particularly for sBCC, enabling efficient clinical decision-making. LC-OCT, on the other hand, outperformed HD-OCT in histologic agreement and iBCC detection.

When comparing the diagnostic performance of available imaging modalities, distinct advantages emerge based on clinical objectives, tumor characteristics, and financial feasibility. Dermoscopy, while affordable (typically under $2000 USD) and accessible, shows relatively lower diagnostic accuracy for BCC subtyping. HFUS and DG-HFUS show improved accuracy and offer the deepest tissue penetration, allowing evaluation of cartilage, muscle, and bone infiltration.^68^^,^^69^ Furthermore, DG-HFUS delivers imaging at a significantly low price (under $20,000 USD), making it a practical option for resource-limited settings. LC-OCT demonstrated the highest diagnostic accuracy and strong histological agreement but shares the high cost (over $100,000 USD) of OCT-based technologies. Similarly, HD-OCT achieved excellent sensitivity for superficial BCCs but faces adoption barriers due to cost. RCM provided reasonable accuracy for superficial and nodular BCCs but is also limited by high cost and the need for specialized operators. Based on these findings, we propose that LC-OCT be prioritized for comprehensive subtyping, HD-OCT for high-sensitivity superficial BCC detection, and DG-HFUS as a cost-effective alternative for initial risk stratification and clinical decision-making.

### Limitations and future directions

Additionally, for certain modalities such as DG-HFUS, D-OCT, LC-OCT, and HD-OCT, only a single qualifying study was identified, with sample sizes ranging from approximately 48 to 81 lesions per modality, which may be insufficient to draw definitive conclusions regarding their diagnostic efficacy. While these modalities show promise for diagnosing and subtyping BCC, limitations remain. Future research should focus on larger, multicenter studies directly comparing these techniques, assessing not only diagnostic accuracy but also clinical impact, cost-effectiveness, and integration into real-world care pathways.

## Conclusion

Novel noninvasive imaging techniques, such as DG-HFUS, HFUS, OCT, LC-OCT, and RCM, show great promise in BCC subtyping by offering higher accuracy than dermoscopy. Among these, HD-OCT and LC-OCT have proven to be the most effective FDA- and EMA-approved imaging modalities for BCC subtyping, and their use may be justified as an alternative to invasive biopsy to confirm low-risk features and direct destructive or topical treatment. However, current studies are limited, and larger, more standardized trials are needed to validate these technologies, optimize clinical workflows, and explore the potential of multimodal approaches for even greater accuracy.

## Conflicts of interest

Dr Wortsman reports royalties from Springer Books, medical writing support from Novartis (unpaid to her), honoraria for lectures from Novartis and UCB, consulting fees from Galderma and unpaid participation in the editorial board of the Journal of Ultrasound in Medicine, Journal of the American Academy of Dermatology International, and Skin, Research, and Technology. Dr Pietkiewicz reports consulting fees from Heine Optotechnik, honoraria for lectures from FotoFinder Systems, unpaid leadership roles as President of the Polish Dermatoscopy Group and Board Member of the International Dermoscopy Society, and receipt of equipment from FotoFinder Systems, Dermlite, and Heine Optotechnik. Drs Boostani, Pellacani, Suppa, Goldust, Cantisani, Lőrincz, Bánvölgyi, Wikonkál, Huss, Avanaki, Paragh, and Kiss have no conflicts of interest to declare.
